# Case Report: Anti-LGI1 encephalitis during FcRn inhibition with efgartigimod for myasthenia gravis: implications for the limitations of IgG recycling blockade

**DOI:** 10.3389/fimmu.2026.1854357

**Published:** 2026-05-18

**Authors:** Yuka Komatsu, Nobuyuki Eura, Yukako Nishimori, Yoshiaki Kakehi, Takao Kiriyama, Kazuma Sugie

**Affiliations:** Department of Neurology, Nara Medical University, Nara, Japan

**Keywords:** ANTI-LGI1 encephalitis, autoimmune encephalitis, efgartigimod, FcRn inhibition, myasthenia gravis, thymoma

## Abstract

**Objectives:**

Anti–leucine-rich glioma-inactivated 1 (LGI1) antibody–associated autoimmune encephalitis is a treatable disorder characterized by subacute cognitive impairment. Efgartigimod is a neonatal Fc receptor (FcRn) antagonist that reduces circulating IgG levels. It is approved for the treatment of generalized myasthenia gravis (MG) and has recently been explored as a therapeutic option for antibody-mediated neurological diseases. We report a patient who developed anti-LGI1 antibody–associated encephalitis during ongoing efgartigimod therapy despite reduced serum IgG levels, raising considerations regarding the mechanisms of central nervous system autoimmunity under FcRn inhibition.

**Methods:**

A 72-year-old woman with thymoma-associated MG receiving efgartigimod was evaluated after presenting with rapidly progressive memory impairment. Clinical assessment included neurological examination, brain magnetic resonance imaging, cerebrospinal fluid analysis, and serological testing for neuronal and paraneoplastic antibodies.

**Results:**

Brain magnetic resonance imaging revealed bilateral medial temporal lobe hyperintensities. Cerebrospinal fluid analysis showed negative LGI1 antibodies and a normal IgG index (0.524), with no evidence suggestive of intrathecal IgG synthesis. Subsequent serological testing confirmed anti-LGI1 antibody positivity, and anti-titin antibodies were also detected. High-dose intravenous methylprednisolone therapy resulted in marked clinical improvement.

**Discussion:**

This case highlights a clinically relevant scenario in which autoimmune encephalitis can arise during FcRn inhibition therapy, suggesting that reduction of circulating IgG alone may be insufficient to prevent central nervous system autoimmunity. The absence of intrathecal IgG synthesis raises the possibility that pathogenic antibodies were predominantly derived from the peripheral compartment. These findings underscore the pharmacodynamic limitations of FcRn inhibition, particularly in the setting of thymoma-associated immune dysregulation.

## Introduction

Autoimmune encephalitis (AE) comprises inflammatory disorders of the central nervous system caused by immune responses directed against neuronal cell-surface or synaptic proteins. Clinically, AE presents with a subacute onset of cognitive impairment, behavioral changes, seizures, and psychiatric symptoms, and early immunotherapy is associated with improved neurological outcomes (1).

Anti–leucine-rich glioma-inactivated 1 (LGI1) antibody–associated encephalitis is a prototypical form of antibody-mediated AE, characterized by subacute cognitive decline, memory disturbance, seizures, and frequently faciobrachial dystonic seizures (FBDS). It is regarded as the second most common subtype of AE after anti–N-methyl-D-aspartate receptor encephalitis (2, 3). Neuroimaging typically demonstrates involvement of the medial temporal lobes, and immunotherapy—including corticosteroids, intravenous immunoglobulin, and plasma exchange—leads to substantial clinical improvement in most patients (3, 4).

Efgartigimod is a neonatal Fc receptor (FcRn) antagonist that reduces circulating immunoglobulin G (IgG) levels by accelerating IgG catabolism and is approved for the treatment of generalized myasthenia gravis (MG). Given its targeted effect on IgG homeostasis, FcRn inhibition has recently emerged as a promising therapeutic strategy for IgG-mediated autoimmune neurological disorders, including AE. Indeed, a recent case series have reported clinical improvement following efgartigimod treatment in patients with AE (5). However, whether suppression of circulating IgG alone is sufficient to prevent the emergence of antibody-mediated autoimmunity within the central nervous system remains uncertain. In particular, data regarding the occurrence of AE during ongoing FcRn inhibition therapy are extremely limited. Here, we describe a patient with thymoma-associated MG who developed anti-LGI1 antibody–associated encephalitis during continued efgartigimod treatment, despite IgG levels being reduced. This case provides insight into the mechanisms of antibody-mediated central nervous system autoimmunity under FcRn inhibition.

## Methods

A 72-year-old woman with thymoma-associated generalized myasthenia gravis (MG) receiving efgartigimod was evaluated for rapidly progressive cognitive impairment. Clinical assessment included neurological examination, standardized cognitive testing (Mini-Mental State Examination and Hasegawa Dementia Scale–Revised), brain magnetic resonance imaging (MRI), electroencephalography, cerebrospinal fluid analysis, and serological testing for neuronal and paraneoplastic antibodies. Treatment decisions were guided by the established diagnostic criteria for autoimmune encephalitis.¹ Written informed consent for publication was obtained from the patient, and the study was approved by the Ethics Committee of Nara Medical University (approval ID: 1837).

## Results

The patient was admitted with rapidly progressive memory impairment evolving over approximately 10 days. She had a long-standing history of thymoma-associated generalized MG. An invasive thymoma (stage IVA) had been surgically resected in 1998 following neoadjuvant chemotherapy, with adjuvant chemotherapy administered postoperatively. MG was diagnosed in 2009. At presentation, MG was clinically stable under treatment with efgartigimod, oral prednisolone (7.5 mg/day), tacrolimus (4 mg/day), and pyridostigmine bromide (60 mg three times daily). Disease severity was moderate but stable (Quantitative Myasthenia Gravis score 10; MG Composite score 11; MG-ADL score 6). Her medical history included chronic empyema. Family history was unremarkable, and she had no history of alcohol consumption or smoking.

**Figure 1:**
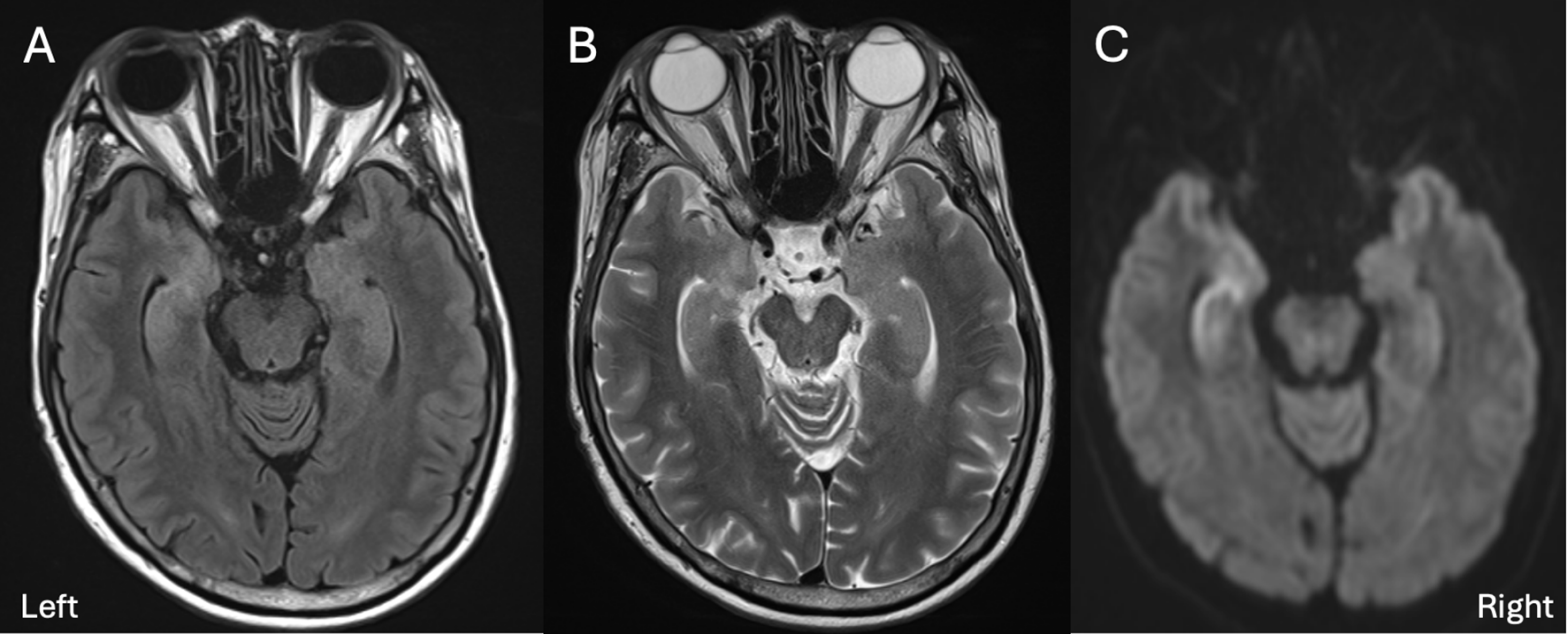
Brain magnetic resonance imaging findings at presentation. Axial fluid-attenuated inversion recovery (FLAIR) image **(A)** demonstrates hyperintense lesions in the bilateral medial temporal lobes, including the hippocampi and amygdalae. The corresponding T2-weighted image **(B)** shows similar signal abnormalities. Diffusion-weighted imaging **(C)** also shows hyperintense lesions in the bilateral medial temporal lobes.

**Figure 2:**
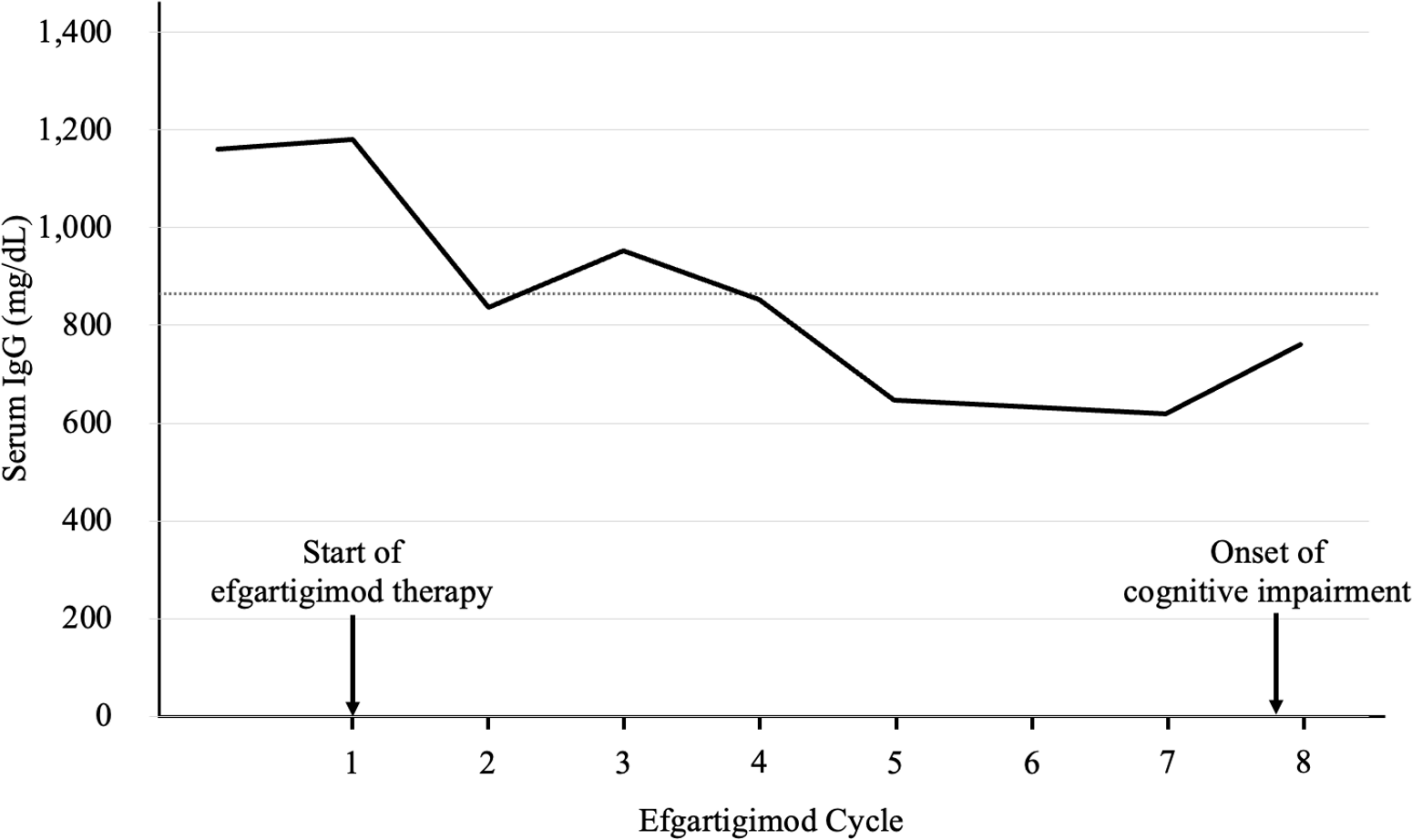
Longitudinal changes in serum immunoglobulin (IgG) levels during efgartigimod treatment. Serum IgG levels measured immediately before each efgartigimod treatment cycle (cycles 1–8) are shown. The dotted horizontal line indicates the lower limit of the institutional reference range for serum IgG (861 mg/dL). Arrows indicate the initiation of efgartigimod therapy and the onset of cognitive impairment.

**Figure 3:**
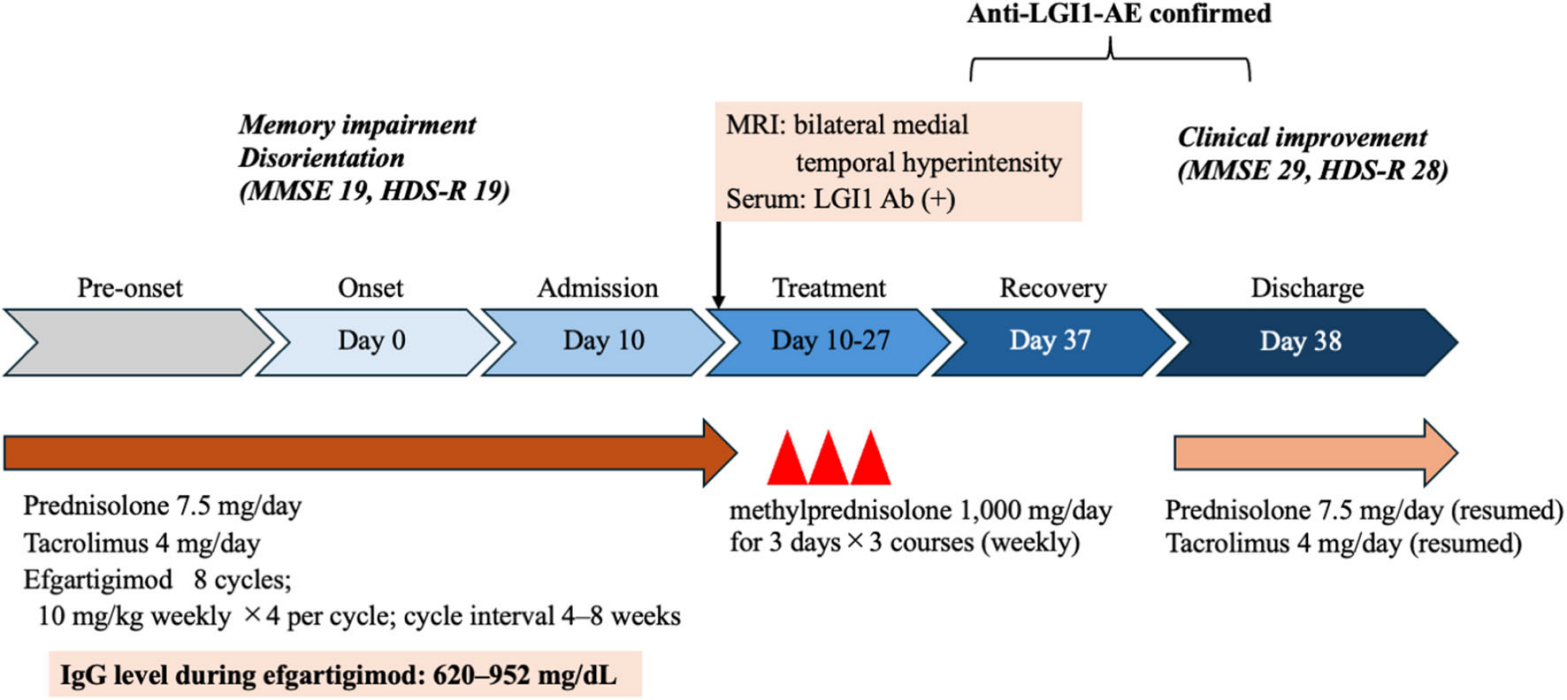
Clinical timeline of the patient. The patient developed memory impairment and disorientation at onset (Day 0). At admission (Day 10), cognitive decline was observed (Mini-Mental State Examination [MMSE] 19, Hasegawa Dementia Scale-Revised [HDS-R] 19), with bilateral medial temporal lobe hyperintensities on MRI and positive serum anti-LGI1 antibodies. Intravenous methylprednisolone was administered (Day 10–27), leading to clinical and cognitive improvement by Day 38 (MMSE 29, HDS-R 28). Oral immunosuppressive therapy was resumed at discharge.

In mid-November 2025, she became unable to recall the names of her cohabiting grandchildren, followed by progressive disorientation and difficulty navigating familiar routes. Brain MRI revealed abnormal signal intensity in the bilateral medial temporal lobes (**Figures 1A–C**), prompting hospital admission. Neurological examination showed mild disturbance of consciousness (Glasgow Coma Scale E4V4M6) and significant cognitive impairment (Mini-Mental State Examination [MMSE] 19; Hasegawa Dementia Scale-Revised [HDS-R] 19), particularly affecting orientation, delayed recall, calculation, and object memory. Cranial nerve examination was unremarkable, with no ptosis, ophthalmoplegia, dysarthria, or dysphagia. Motor examination revealed mild weakness of neck flexion (Medical Research Council grade 4−) and extension (grade 3). No FBDS, other seizures, or involuntary movements were observed. There were no abnormalities in sensory, cerebellar, or autonomic examinations. Routine laboratory tests were normal. Serum IgG level was reduced to 761 mg/dL (reference range 861-1,747 mg/dL) and serum sodium level was 141 mEq/L (**Table 1**). No metabolic abnormalities, including hypoglycemia, hypotension, or hypoxia, that could account for the cognitive impairment were identified (**Table 2**). Immunological testing demonstrated positivity for anti–acetylcholine receptor antibodies (16 nmol/L) and anti-titin antibodies, while anti–glutamic acid decarboxylase antibodies, anti-thyroglobulin antibodies, and anti–thyroid peroxidase antibodies were negative. Serum anti-LGI1 antibodies were detected using a commercial cell-based assay (indirect immunofluorescence) performed by a reference laboratory (Cosmic Corporation, Tokyo, Japan). Cerebrospinal fluid analysis showed a cell count of 5/μL with normal protein levels (39.2 mg/dL). Cerebrospinal fluid analysis showed negative LGI1 antibodies, a normal IgG index (0.524), and no evidence suggestive of intrathecal IgG synthesis. Bacterial cultures were negative, and polymerase chain reaction testing for herpes simplex virus and varicella-zoster virus was negative. MRI demonstrated bilateral hippocampal and amygdalar hyperintensities on fluid-attenuated inversion recovery (FLAIR) and T2-weighted images. Electroencephalography showed diffuse theta–alpha activity without epileptiform discharges. Contrast-enhanced computed tomography demonstrated progressive pleural dissemination of thymoma. Four days before admission, the patient had received the first infusion of the eighth cycle of efgartigimod (**Figure 2**). To maintain MG stability, the remaining infusions were administered during hospitalization. Given the subacute cognitive decline and medial temporal lobe involvement, the patient fulfilled the diagnostic criteria for possible AE (**Figure 3**) (1). High-dose intravenous methylprednisolone (1,000 mg/day for 3 consecutive days) was administered for three courses, with concomitant acyclovir until viral etiologies were excluded. Cognitive function gradually improved, with MMSE and HDS-R scores increasing to 29 and 28, respectively. Subsequent serological testing confirmed anti-LGI1 antibody positivity, establishing the diagnosis of anti-LGI1 antibody–associated AE. Maintenance therapy for MG was continued, and the patient was discharged on hospital day 39 with marked clinical improvement. Multidisciplinary consultation planned systemic chemotherapy for progressive thymoma.

**Table 1:** Summary of laboratory and paraclinical findings at presentation.

Category	Parameter	Result
Blood tests	Sodium	141 mEq/L
	Serum IgG	761 mg/dL
Immunology	Anti-LGI1 Ab (serum)	Positive
	Anti-LGI1 Ab (CSF)	Negative
	Anti-AChR Ab	16 nmol/L
	Anti-titin Ab	Positive
	Anti-GAD Ab	<5.0 U/mL
	Anti-thyroglobulin Ab	Negative
	Anti-TPO Ab	Negative
CSF	Cell count	5/μL
	Protein	39.2 mg/dL
	IgG index	0.524
	HSV PCR	Negative
	VZV PCR	Negative
	Bacterial culture	Negative
Imaging	Brain MRI	Bilateral medial temporal hyperintensity
Neurophysiology	EEG	Diffuse theta–alpha activity

Laboratory and paraclinical data obtained at disease onset are summarized. Serum and cerebrospinal fluid (CSF) antibody testing included anti-leucine-rich glioma-inactivated 1 (LGI1) antibodies. Brain magnetic resonance imaging (MRI) revealed bilateral medial temporal hyperintensity. Electroencephalography (EEG) showed diffuse theta–alpha activity.

CSF, cerebrospinal fluid; LGI1, leucine-rich glioma-inactivated 1; AChR, acetylcholine receptor; GAD, glutamic acid decarboxylase; TPO, thyroid peroxidase; HSV, herpes simplex virus; VZV, varicella-zoster virus; PCR, polymerase chain reaction; MRI, magnetic resonance imaging; EEG, electroencephalography.

**Table 2:** Differential diagnoses considered and rationale for exclusion.

Diagnosis	Supporting features	Findings against diagnosis
Herpes simplex encephalitis	Subacute cognitive decline, medial temporal involvement	Negative HSV PCR (CSF), no fever, good response to steroids
Other autoimmune encephalitis (e.g., anti-NMDAR)	Subacute cognitive impairment	Negative neuronal antibody panel except LGI1
Hashimoto encephalopathy	Cognitive impairment	Negative anti-thyroid antibodies, lack of typical features
Paraneoplastic limbic encephalitis	Thymoma, subacute symptoms	No classical onconeural antibodies were detected, although anti-titin antibodies were positive
Metabolic/toxic encephalopathy	Altered mental status	Normal laboratory findings, no metabolic derangement
Neurodegenerative disease (e.g., Alzheimer’s disease)	Memory impairment	Rapid progression, MRI findings, response to immunotherapy

A structured differential diagnostic approach was applied based on clinical presentation, neuroimaging findings, cerebrospinal fluid (CSF) analysis, and serological testing. For each condition, features supporting the diagnosis and key findings leading to its exclusion are summarized. The patient’s favorable response to immunotherapy further supported an autoimmune etiology. Taken together, these findings supported the diagnosis of LGI1 antibody-associated autoimmune encephalitis.

CSF, cerebrospinal fluid; HSV, herpes simplex virus; MRI, magnetic resonance imaging; NMDAR, N-methyl-D-aspartate receptor.

## Discussion

This case demonstrates the development of anti–LGI1 antibody–associated AE during ongoing FcRn inhibition therapy with efgartigimod in a patient with thymoma-associated MG, despite reduced serum IgG levels. In our patient, cerebrospinal fluid (CSF) analysis showed negative LGI1 antibodies and a normal IgG index, with no clear evidence of intrathecal IgG synthesis.

Although LGI1 antibodies are frequently detected in both serum and CSF, discordant findings—particularly CSF-negative and serum-positive cases—have been consistently reported (3, 6). In cohort studies, LGI1 antibodies are more frequently detected in serum, and some patients show CSF negativity despite typical clinical features (3). This discrepancy may partly reflect differences in assay sensitivity, as more advanced cell-based assays have demonstrated improved detection rates, suggesting that some “CSF-negative” cases may be due to methodological limitations rather than true absence of intrathecal antibodies (7). Clinically, such patients are largely indistinguishable from those with CSF positivity, supporting the notion that serum positivity alone can be diagnostically meaningful in the appropriate context (6). Furthermore, LGI1 antibodies are considered pathogenic, as they disrupt LGI1–ADAM22/23 interactions and alter synaptic AMPA receptor function, leading to neuronal hyperexcitability (8, 9). Taken together, these findings support that serum LGI1 antibody positivity, even without CSF positivity, is unlikely to be incidental in this case.

Consistent with this interpretation, the encephalitis was highly responsive to corticosteroid therapy, further supporting the diagnosis of LGI1 antibody–associated encephalitis (3, 10).

Efgartigimod reduces circulating IgG by accelerating FcRn-mediated IgG catabolism and has expanded therapeutic options for antibody-mediated neurological diseases (5). However, FcRn inhibition primarily affects IgG recycling and does not suppress autoreactive B cells, eliminate long-lived plasma cells, or inhibit ongoing antibody production. Recent studies have demonstrated that efgartigimod exerts modulatory effects on B-cell differentiation and immune homeostasis, influencing the dynamics of the antibody pool beyond simple IgG clearance (11). However, the extent to which these effects suppress ongoing pathogenic autoantibody production remains unclear, particularly in conditions associated with persistent immune activation. Consequently, pathogenic IgG may persist despite reduced total IgG levels. This distinction is critical for understanding the pharmacodynamic limitations of FcRn blockade. In this context, the present case suggests that reduction of circulating IgG alone may be insufficient to prevent the emergence of central nervous system autoimmunity.

The absence of intrathecal IgG synthesis in this case may suggest a predominant contribution from peripherally derived antibodies; however, compartmentalized or low-level intrathecal antibody production cannot be excluded. In addition (12), FcRn is expressed at the blood–brain barrier and is thought to contribute to IgG transport between the central nervous system and peripheral circulation (13). Inhibition of this pathway may therefore have complex and incompletely understood effects on antibody dynamics across compartments, potentially limiting the impact of peripheral IgG reduction on central nervous system inflammation (1, 14).

Importantly, this patient had thymoma-associated MG with anti-titin antibody positivity and radiologically progressive pleural dissemination of thymoma, suggesting persistent active disease and systemic immune dysregulation (15). Although anti-LGI1 encephalitis is not classically categorized as a paraneoplastic neurological syndrome, thymoma-associated breakdown of immune tolerance may facilitate the emergence of multiple autoantibody responses, including those targeting central nervous system antigens. In this context, ongoing antigenic stimulation from thymoma may have contributed to the development of AE despite pharmacological IgG reduction.

This report has several limitations. As a single case, a causal relationship between FcRn inhibition and the development of AE cannot be established. In addition, IgG subclass distribution and longitudinal antibody titers were not evaluated, and continuation of efgartigimod during the acute phase precludes assessment of its direct impact on disease onset and progression. Longitudinal measurements of LGI1 antibody titers were not available in this case. Furthermore, neurofilament light chain (NfL) levels were not evaluated, although they may provide useful information regarding disease severity and long-term cognitive outcomes (16).

In summary, this case illustrates the pharmacodynamic limitations of IgG recycling blockade in the setting of ongoing autoantibody production and immune dysregulation. These findings suggest that, in selected patients with AE, therapies targeting antibody production—such as glucocorticoids or B cell–depleting strategies— may be required in addition to FcRn inhibition to achieve adequate disease control. Although such strategies may be considered in patients with persistent autoantibody production or underlying immune dysregulation, their efficacy and safety in combination with FcRn inhibitors remain to be established and warrant further investigation.

## Patient perspective

The patient reported a noticeable improvement in memory and daily functioning following corticosteroid therapy. She was able to resume her usual activities after discharge and expressed satisfaction with the treatment outcome.

## Data Availability

The raw data supporting the conclusions of this article will be made available by the authors, without undue reservation.

## Glossary

AE: autoimmune encephalitis
LGI1: leucine-rich glioma-inactivated 1
FcRn: neonatal Fc receptor
MG: myasthenia gravis
MRI: magnetic resonance imaging
FLAIR: fluid-attenuated inversion recovery
CSF: cerebrospinal fluid
EEG: electroencephalography
MMSE: Mini-Mental State Examination
HDS-R: Revised Hasegawa Dementia Scale
QMG: Quantitative Myasthenia Gravis
MG-ADL: Myasthenia Gravis Activities of Daily Living
